# Arabidopsis HD-Zip II proteins regulate the exit from proliferation during leaf development in canopy shade

**DOI:** 10.1093/jxb/ery331

**Published:** 2018-09-15

**Authors:** Monica Carabelli, Marco Possenti, Giovanna Sessa, Valentino Ruzza, Giorgio Morelli, Ida Ruberti

**Affiliations:** 1 Institute of Molecular Biology and Pathology, National Research Council, Rome, Italy; 2 Research Centre for Genomics and Bioinformatics, Council for Agricultural Research and Economics (CREA), Rome, Italy

**Keywords:** Arabidopsis, HD-Zip II transcription factors, HFR1/SICS1, leaf development, light quality changes, shade avoidance response

## Abstract

The shade avoidance response is mainly evident as increased plant elongation at the expense of leaf and root expansion. Despite the advances in understanding the mechanisms underlying shade-induced hypocotyl elongation, little is known about the responses to simulated shade in organs other than the hypocotyl. In Arabidopsis, there is evidence that shade rapidly and transiently reduces the frequency of cell division in young first and second leaf primordia through a non-cell-autonomous mechanism. However, the effects of canopy shade on leaf development are likely to be complex and need to be further investigated. Using combined methods of genetics, cell biology, and molecular biology, we uncovered an effect of prolonged canopy shade on leaf development. We show that persistent shade determines early exit from proliferation in the first and second leaves of Arabidopsis. Furthermore, we demonstrate that the early exit from proliferation in the first and second leaves under simulated shade depends at least in part on the action of the Homeodomain-leucine zipper II (HD-Zip II) transcription factors ARABIDOPSIS THALIANA HOMEOBOX2 (ATHB2) and ATHB4. Finally, we provide evidence that the ATHB2 and ATHB4 proteins work in concert. Together the data contribute new insights on the mechanisms controlling leaf development under canopy shade.

## Teaser

Prolonged shade determines early exit from proliferation in the Arabidopsis leaf through the concerted action of ATHB2 and ATHB4 HD-Zip II proteins.

## Introduction

In dicots, plant growth is controlled by internal pathways that are strongly influenced by the environment. The effects of light on shoot growth are particularly interesting, because both light quantity and quality dynamically change during the day (Smith, 1982).

For instance, the reduction in the red/far red (R/FR) ratio of light acts as a signal that triggers the shade avoidance response, causing profound changes in stem and petiole elongation and leaf area growth in the angiosperms that have evolved the capacity to avoid shade. In contrast, shade-tolerant plants have adapted their photosynthesis to function optimally under low-light conditions. These plants are therefore capable of long-term survival under a canopy shade. In Arabidopsis, a typical shade-avoiding plant, the shade avoidance response consists of hypocotyl and petiole elongation and reduction of leaf lamina growth at the early stage of seedling development, and of elevation (hyponasty) and elongation of leaf petioles in older plants (Franklin, 2008; Casal, 2012).

The shade avoidance response occurs when plants grow close to each other, because the light environment is strongly enriched in FR by the light reflected by neighbour plants, and under foliar or canopy shade. In fact, light absorption by photosynthetic pigments occurs mainly in the blue (B) and R (400–500 nm and 600–700 nm, respectively) whereas most of the FR (700–750 nm) is reflected or transmitted by plant tissues. Hence, under canopy shade, both the amount of photosynthetically active radiation (PAR) and the R/FR ratio are significantly reduced (Smith, 1982; Martinez-Garcia *et al.*, 2010; Casal, 2013; Roig-Villanova and Martínez-García, 2016; Fiorucci and Fankhauser, 2017). Although the leading signal triggering the shade avoidance response is the low R/FR perceived through the phytochrome (phy) systems, the reduced R and B irradiance and the blue/green ratio are also important for the physiological and developmental responses (Casal, 2013; Pedmale *et al.*, 2016; Roig-Villanova and Martínez-García, 2016). Indeed, a decrease in the blue/green ratio causes stem elongation in Arabidopsis similar to a decrease in R/FR ratio (Sellaro *et al.*, 2010), although operating through different mechanisms (Pedmale *et al.*, 2016). The internode elongation seen in plants grown under a low R/FR ratio and/or low PAR appears to be mediated by changes in endogenous plant hormone levels and sensitivity (Morelli and Ruberti, 2000; Müller-Moulé *et al.*, 2016; Ballaré and Pierik, 2017). It has been suggested that hypocotyl elongation caused by neighbour detection is promoted primarily by the production of auxin (Casal, 2013). In contrast, under canopy shade, the system operates with less auxin but with an increased sensitivity to the hormonal signal, probably through a differential activity of PHYTOCHROME INTERACTING FACTOR4 (PIF4), PIF5, and LONG HYPOCOTYL IN FAR RED 1/SLENDER IN CANOPY SHADE 1 (HFR1/SICS1) (Hersch *et al.*, 2014). The PIF proteins are basic helix–loop–helix (bHLH) transcription factors that interact with phyB through the conserved N-terminal sequence, the active phyB-binding motif (Leivar and Quail, 2011). *HFR1/SICS1* encodes an atypical bHLH protein, and acts as a HLH inhibitor (Hornitschek *et al.*, 2009). PIF1, PIF3, and PIF7, like PIF4, PIF5, and HFR1/SICS1, have been implicated in shade avoidance (Sessa *et al.*, 2005; Lorrain *et al.*, 2008; Hornitschek *et al.*, 2012; Leivar *et al.*, 2012; Li *et al.*, 2012; de Wit *et al.*, 2015). Low R/FR-induced elongation response is attenuated in the *pif4 pif5* double mutant and, even more, in *pif1 pif3 pif4 pif5* quadruple and *pif7* mutants (Lorrain *et al.*, 2008; Leivar *et al.*, 2012; Li *et al.*, 2012). In contrast, transgenic seedlings expressing elevated levels of PIF4 and PIF5 display constitutively long hypocotyls and petioles (Lorrain *et al.*, 2008). In addition, PIF4, PIF5, and PIF7 have been shown to bind directly the promoters of YUCCA8 and YUCCA9 (Hornitschek *et al.*, 2012; Li *et al.*, 2012), auxin biosynthetic genes essential for shade-induced elongation growth (Müller-Moulé *et al.*, 2016).

In Arabidopsis, the shade avoidance response is regulated by a balance of positive and negative factors which, on one hand, ensures a rapid reshaping of the plant towards a light environment more favourable for growth, and, on the other hand, avoids an exaggerated reaction to low R/FR light (Sessa *et al.*, 2005; Bou-Torrent *et al.*, 2008; Crocco *et al.*, 2010; Galstyan *et al.*, 2011; Hao *et al.*, 2012; Ciolfi *et al.*, 2013). Very recently it has been shown that PIF proteins directly suppress the expression of miR156, a negative regulator of the *SQUAMOSA-PROMOTER BINDING PROTEIN-LIKE* (*SPL*) family of genes (Xie *et al.*, 2017). As the *SPL* genes regulate several aspects of plant development, including leaf initiation rate, branching, vegetative phase change, and flowering time (Xu *et al.*, 2016), the findings reported by Xie and co-authors imply that important aspects of the shade avoidance response are positively regulated through the PIF/miR156/SPL module (Xie *et al.*, 2017).

Among the negative regulators of the shade avoidance response is HFR1/SICS1. *HFR1/SICS1* is rapidly induced by low R/FR, and evidence exists that it is a direct target of PIF5 (Sessa *et al.*, 2005; Hornitschek *et al.*, 2009). Persistence of low R/FR results in HFR1/SICS1 accumulation and formation of non-functional heterodimers with PI4 and PIF5 (Sessa *et al.*, 2005; Hornitschek *et al.*, 2009). Accordingly, a number of genes rapidly and transiently induced by low R/FR, including *ATHB2*, are up-regulated in *hfr1/sics1* mutants upon prolonged shade exposure (Sessa *et al.*, 2005; Ruzza *et al.*, 2014). *ATHB2* induction by low R/FR does not require *de novo* protein synthesis (Roig-Villanova *et al.*, 2006), and is reduced in loss-of-function *pif* mutants (*pif1 pif3*; *pif4 pif5*; and *pif7*; Lorrain *et al.*, 2008; Hornitschek *et al.*, 2009; Leivar *et al.*, 2012; Li *et al.*, 2012). Furthermore, it has been shown that *ATHB2* is recognized *in vivo* by PIF5 (Hornitschek *et al.*, 2012). Loss-of-function *athb2* mutants display diminished hypocotyl elongation in low R/FR with respect to wild-type seedlings (Carabelli *et al.*, 2013), whereas the phenotypes of plants overexpressing ATHB2 in high R/FR are reminiscent of those displayed by the wild type grown in low R/FR, indicating a role for this HD-Zip protein in the regulation of the shade avoidance response (Steindler *et al.*, 1999).

*ATHB2* is a member of the HD-Zip II family consisting of 10 genes, five of which [*ATHB2, HOMEOBOX ARABIDOPSIS THALIANA* (*HAT1*), *HAT2*, *ATHB4*, and *HAT3*] are regulated by changes in light quality that induce the shade avoidance response (Ciarbelli *et al.*, 2008). Elevated levels of *HAT1*, *HAT2*, *HAT3*, and *ATHB4* result in phenotypes analogous to those caused by ATHB2 overexpression (Sawa *et al.*, 2002; Ciarbelli *et al.*, 2008; Sorin *et al.*, 2009; Ruberti *et al.*, 2012; Ruzza *et al.*, 2014), further indicating a redundant function of these transcription factors in shade avoidance. Interestingly, homologue genes have been found to be up-regulated in monocotyledonous and dicotyledonous plants exposed to low R/FR light, suggesting a conserved function of HD-Zip II transcription factors through evolution (Ueoka-Nakanishi *et al.*, 2011; Chitwood *et al.*, 2015; Wang *et al.*, 2016).

Despite the significant advances over the last decade in understanding the mechanisms underlying shade-induced hypocotyl elongation (Casal, 2013), little is known about the cellular and molecular responses to low R/FR in the leaf. Previous studies in Arabidopsis have reported that light treatments that simulate neighbour detection or canopy shade promote petiole elongation and reduce leaf lamina growth (Kozuka *et al.*, 2005; Carabelli *et al.*, 2007; Kozuka *et al.*, 2010; Sasidharan *et al.*, 2010; de Wit *et al.*, 2015; Roig-Villanova and Martínez-García, 2016). Under simulated canopy shade, we provide evidence that low R/FR rapidly and transiently reduces the frequency of cell division in first leaf primordia through a non-cell-autonomous mechanism (Carabelli *et al.*, 2007, 2008). However, the effects of canopy shade on leaf development are likely to be complex and need to be further investigated. Here, we show that prolonged low R/FR determines early exit from proliferation in the leaf and that this process requires the HD-Zip II transcription factors ATHB2 and ATHB4.

## Materials and methods

### Mutant, transgenic, and marker lines, and plant growth

The wild-type strain used was *Arabidopsis thaliana* (L.) Heynh. var. Columbia (Col-0). Other lines used were: *athb2-1* (Khanna *et al.*, 2006), *athb2-2* (Turchi *et al.*, 2013), *athb2-3* (Turchi *et al.*, 2013), *athb4-1* (Sorin *et al.*, 2009), *athb4-3* (Turchi *et al.*, 2013), *athb2-1 athb4-1* (Turchi *et al.*, 2013), *hfr1-4/sics1-1* (Sessa *et al.*, 2005), *hfr1-5/sics1-2* (Sessa *et al.*, 2005), ATHB2::ATHB2:GUS (Turchi *et al.*, 2013), *hfr1-4/sics1-1* ATHB2::ATHB2:GUS (Ruzza *et al.*, 2014), ATHB8::GUS (Baima *et al.*, 1995), CYCLINB1;1:GUS (CYCB1;1:GUS) (Cólon-Carmona *et al.*, 1999; Kang and Dengler, 2002). Plants were grown as previously described (Sessa *et al.*, 2005). Light outputs in High R/FR_High PAR_, Low R/FR_Low PAR_, and High R/FR_Low PAR_ were as reported by Ciolfi *et al.* (2013).

### Genetic analysis

To generate *athb2-1 hfr1-4/sics1-1*, *athb4-1 hfr1-4/sics1-1*, and *athb2-2 athb4-1*, the following crosses were performed: *athb2-1*×*hfr1-4/sics1-1*, *athb4-1*×*hfr1-4/sics1-1*, and *athb2-2*×*athb4-1*. Double mutants were selected in F_2_ by phenotyping and PCR genotyping, using the primers previously described (Sessa *et al.*, 2005; Turchi *et al.*, 2013). The double mutants were reanalysed in F_3_ by phenotyping and genotyping. Homozygosity of the CYCB1;1:GUS (β-glucuronidase) reporter in *athb2-2* and *athb2-3* was determined by unanimous GUS staining of all seedlings tested (*n* ≥30).

### Gene constructs and transformation

35S::ATHB4:GFP (green fluorescent protein) was constructed as follows: the coding sequence (cds) of ATHB4 (TAIR AT2G44910), excluding the stop codon, was amplified with Gateway™ ends and cloned into pDONR-201. Subsequently, the ATHB4 cds was transferred in pK7FWG2 (Curtis and Grossniklaus, 2003). 35S::ATHB4:GFP was then transferred to *Agrobacterium tumefaciens* GV3101RK and Col-0 plants were transformed as described (Steindler *et al.*, 1999).

### Phenotype analysis and microscopy

Leaves were cleared according to the protocol previously described (Weigel and Glazebrook, 2002). Cleared samples were excised under an MZ8 binocular microscope (Leica, Germany), and then analysed under dark-field optics or with differential interference contrast (DIC) optics, with an Axioskop 2 plus binocular microscope (Zeiss, Germany). Images were taken with a Coolpix 990 digital camera (Nikon Corp., Japan). To determine the mean leaf area, at least 10 samples were measured with the NIH Image Analysis Software [Research Services Branch (RSB) of the National Institute of Mental Health (NIMH), USA, http://rsb.info.nih.gov/ij/]. *T*-test statistical analysis was performed using QuickCalcs Online Calculators for Scientists (GraphPad Software, Inc. http://graphpad.com/quickcalcs/).

Adaxial subepidermal cells were analysed in cleared young leaves. Cells were viewed with an Axioskop 2 plus binocular microscope equipped with DIC optics, and photographed with the Coolpix 990 digital camera. In young leaves, the degree of cell differentiation and the mean cell area were determined in specific regions of the blade, as indicated. A distal location from the base of the leaf was recognized in the upper part of the leaf blade, inside the first loop of the secondary vein in the procambial or differentiated stage; a proximal region was determined close to the margin of the basal part of the leaf. To determine the mean cell area, 100 cells were measured in the indicated regions of 10 leaves (Horiguchi *et al.*, 2005). Means were compared with *t*-test analysis (http://graphpad.com/quickcalcs/).

### Histochemical detection of GUS activity

For histochemical detection of GUS activity, whole seedlings were treated as previously described (Scarpella *et al.*, 2004; Carabelli *et al.*, 2007). Incubation was 8, 3, and 5 h for ATHB2::ATHB2:GUS, ATHB8::GUS, and CYCB1;1:GUS, respectively. For microscopy, samples were cleared according to the protocol previously described (Weigel and Glazebrook, 2002) and viewed with an Axioskop 2 plus binocular microscope equipped with DIC optics; images were taken with the Coolpix 990. Expression pattern distributions of CYCB1;1:GUS in wild-type and mutant leaves were compared using a contingency table followed by Fisher’s exact test (http://www.physics.csbsju.edu/stats/contingency.html).

### Cyclin index determination

In cleared leaves the total cell number and the number of cells with GUS activity in the adaxial subepidermal layer were counted at the proximal region along the proximo-distal axis of the blade by photographing leaves under DIC optics. A 0.01 mm^2^ area was recognized in the proximal region close to the margin of the basal part of the leaf blade. The total cell number and the number of cells with GUS activity were counted, and expressed as the cyclin index (no. of cells with GUS activity/no. of total cells×100) (Donnelly *et al.*, 1999). The data are presented in the form of box-and-whiskers plots, generated by means of the software available at http://plot.ly. A ‘boxplot’ function with default parameters was used (Carabelli *et al.*, 2007).

### Real-time PCR

For gene expression analysis, mRNA purification, cDNA synthesis, and quantitative real-time PCR (RT-qPCR) were performed as previously described (Ciarbelli *et al.*, 2008). Primers and Universal Probe Library (UPL) probes for RT-qPCR analyses are described in Supplementary Table S1 at *JXB* online. Statistical analyses were performed on log-transformed relative expression ratio values as described by Rieu and Powers (2009). The relative transcript abundance of each gene was normalized to the Col-0 level in high R/FR. Subsequent to data standardization (Willems *et al.*, 2008), one-way ANOVA followed by a Tukey’s post-hoc test was used to assess differences among means (Prism 5, GraphPad Software, CA, USA).

## Results

### Prolonged shade determines early exit from proliferation in the leaf

**Fig. 1.**
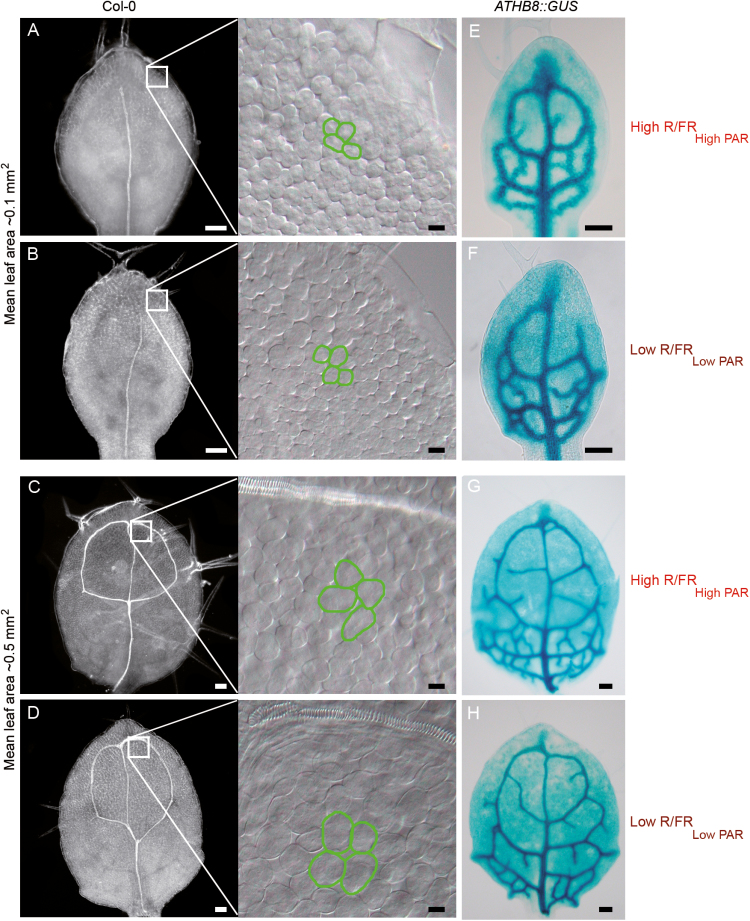
Shade affects adaxial subepidermal cell expansion and vein pattern formation in the leaf. (A–D) Dark-field images of cleared first and second leaves of Col-0 seedlings grown for 7 d (A) and 8.5 d (C) in High R/FR_High PAR_ (High R/FR_High PAR_), or for 4 d in High R/FR_High PAR_ and subsequently for 4 d (B) and 6 d (D) in Low R/FR_Low PAR_ (Low R/FR_Low PAR_), respectively. The insets show a paradermal view of adaxial subepidermal cells; the borders of a few cells have been highlighted manually with a green line. (E–H) Histochemical localization of GUS activity in the first and second leaves of ATHB8::GUS seedlings grown as in (A–D). First and second leaf area (mean ±SE): (A) 0.10 ± 0.005 mm^2^; (B) 0.09 ± 0.003 mm^2^; (C) 0.52 ± 0.007 mm^2^; (D) 0.50 ± 0.003 mm^2^; (E) 0.09 ± 0.004 mm^2^; (F) 0.10 ± 0.003 mm^2^; (G) 0.51 ± 0.022 mm^2^; (H) 0.50 ± 0.024 mm^2^. Scale bars: (A–H) 100 μm; insets, 10 μm.

We have previously shown that leaves grown in simulated shade (Low R/FR_Low PAR_) are significantly smaller than those grown in simulated sun (High R/FR_High PAR_) and that cell number, not cell size, contributes to the reduction of leaf area under Low R/FR_Low PAR_. We have also provided evidences that Low R/FR _Low PAR_ rapidly and transiently reduces the frequency of cell division in first leaf primordia (Carabelli *et al.*, 2007). Here, we reasoned that if the only effect of shade on leaf development is to induce a transient arrest of cell division in young leaf primordia, at later developmental stages leaves of equal area grown in High R/FR_High PAR_ and Low R/FR_Low PAR_ environments should show no significant morphological difference. To test this, first and second leaves of different ages but the same area in High R/FR_High PAR_ (7- and 8.5-day-old seedlings; Supplementary Fig. S1) and Low R/FR_Low PAR_ (8- and 10-day old seedlings; Supplementary Fig. S1) were analysed by examining adaxial subepidermal cells that are good markers of cell differentiation and leaf expansion. No significant difference was observed in the adaxial subepidermal ground meristem cells of leaves with an area of ~0.1 mm^2^ (see legend to Fig. 1 for mean leaf area) in High R/FR_High PAR_ and Low R/FR_Low PAR_ (Fig. 1A, B, insets; mean cell area of distal leaf region 73.7 ± 1.8 μm^2^ and 69.5 ± 1.6 μm^2^ in High R/FR_High PAR_ and Low R/FR_Low PAR_, respectively). Consistent with this, the ground meristem cells showed the same morphological features: rectangular shape, mitotic activity, and absence of airspaces (Fig. 1A, B, insets and data not shown). In contrast, adaxial subepidermal cells of leaves with an area of ~0.5 mm^2^ (see legend to Fig. 1 for mean leaf area) were larger throughout the organ in Low R/FR_Low PAR_ than in High R/FR_High PAR_ (Fig. 1C, D, insets; mean cell area of distal leaf region 187.9 ± 4.3 μm^2^ and 218.9 ± 6.7 μm^2^ in High R/FR_High PAR_ and Low R/FR_Low PAR_, respectively, *P*<0.0002; mean cell area of proximal leaf region 34.4 ± 0.8 μm^2^ and 48.1 ± 1.4 μm^2^ in High R/FR_High PAR_ and Low R/FR_Low PAR_, respectively, *P*<0.0001). In the distal region of the organ, cells were also more vacuolated and separated by larger airspaces in Low R/FR_Low PAR_, suggesting that mesophyll cell differentiation is initiated earlier in Low R/FR_Low PAR_ than in High R/FR_High PAR_ (Fig. 1C, D, insets and data not shown).

To investigate further the effects of shade on leaf development, the complexity of the vascular system was analysed. At the 0.1 mm^2^ stage, first and second leaves in High R/FR_High PAR_ and Low R/FR_Low PAR_ have both formed the first-order vein (Fig. 1A, B). Moreover, histochemical localization of GUS activity in 0.1 mm^2^ first and second leaves of seedlings expressing the ATHB8::GUS marker (Baima *et al.*, 1995) showed no difference in the number of GUS-stained procambial strands in High R/FR_High PAR_ and Low R/FR_Low PAR_ (Fig. 1E, F; mean number of GUS-stained strands 14.9 ± 0.7 and 13.9 ± 0.5 in High R/FR_High PAR_ and Low R/FR_Low PAR_, respectively, *n*=10 leaves). At the 0.5 mm^2^ stage, first and second leaves in High R/FR_High PAR_ and Low R/FR_Low PAR_ have both formed the vascular loop of second-order veins (Fig. 1C, D). However, they have developed more procambial strands of higher order in High R/FR_High PAR_ than in Low R/FR_Low PAR_, as deduced by the expression pattern of the ATHB8::GUS marker (Fig. 1G, H; mean number of GUS-stained strands 26.5 ± 0.7 and 17.8 ± 0.9 in High R/FR_High PAR_ and Low R/FR_Low PAR_, respectively, *n*=10 leaves, *P*<0.0001).

This finding further suggests that mesophyll cell differentiation is initiated earlier in Low R/FR_Low PAR_ than in High R/FR_High PAR_. In fact, there is evidence that the Arabidopsis vein pattern is not inherently determinate, but arises through reiterative initiation of new pre-procambial branches until this process becomes terminate by the differentiation of mesophyll (Scarpella *et al.*, 2004).

**Fig. 2.**
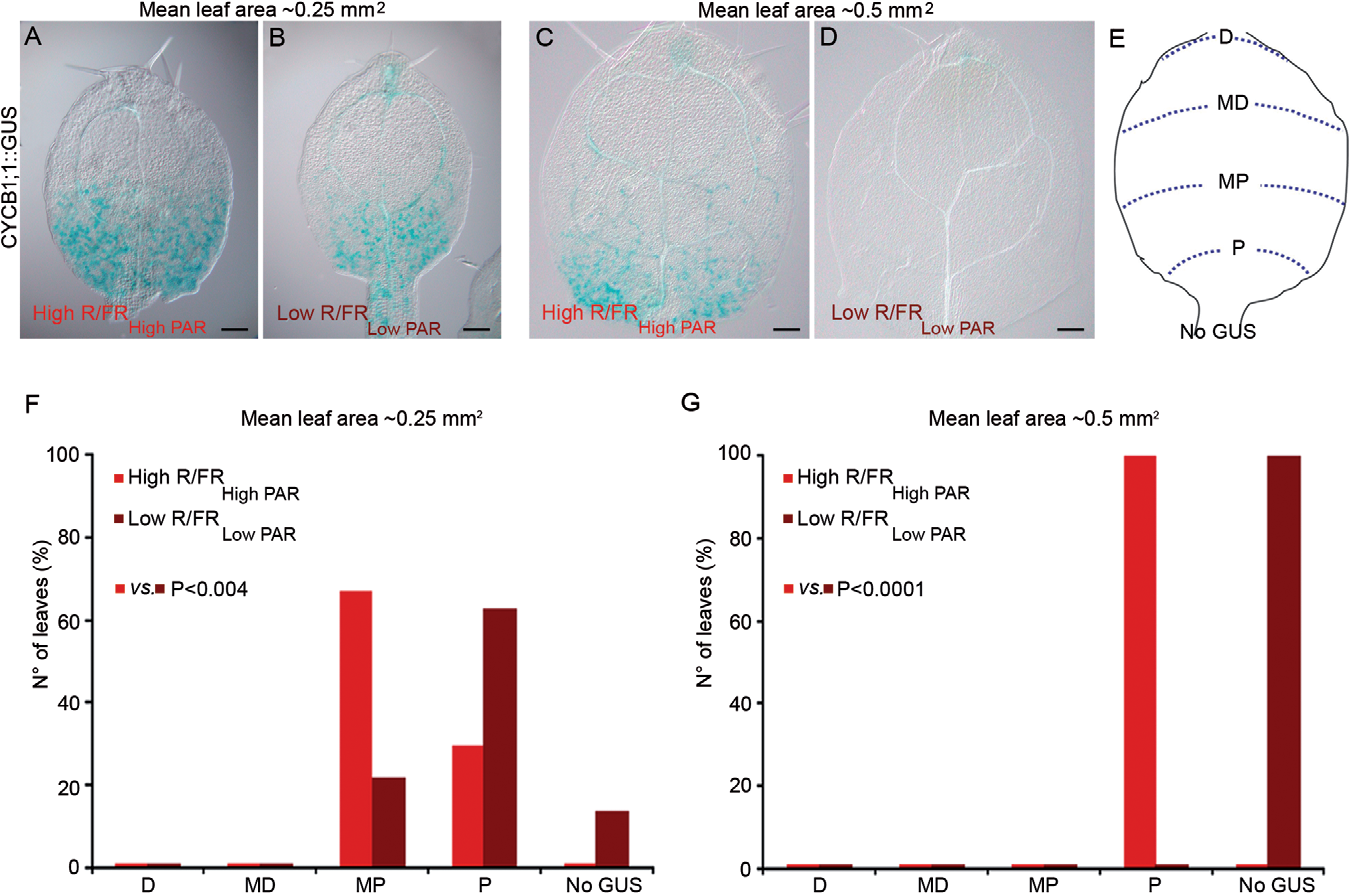
Shade affects the spatial expression pattern of the CYCB1;1:GUS marker in the leaf. (A–D) Histochemical localization of GUS activity in the adaxial subepidermal cells of the first and second leaves of CYCB1;1:GUS seedlings grown in a light/dark cycle (16/8 h) for 7.5 d (A) and 8.5 d (C) in High R/FR_High PAR_ (High R/FR_High PAR_), or for 4 d in High R/FR_High PAR_ and subsequently for 5 d (B) and 6 d (D) in Low R/FR_Low PAR_ (Low R/FR_Low PAR_), respectively. First and second leaf area (mean ±SE): (A) 0.25 ± 0.011 mm^2^; (B) 0.24 ± 0.009 mm^2^; (C) 0.53 ± 0.013 mm^2^; (D), 0.54 ± 0.012 mm^2^. Scale bars: 100 μm. (E–G) Quantification of the CYCB1;1:GUS expression pattern in different regions of the first and second leaf blade. For each condition, at least 10 leaves were analysed and divided into four GUS expression patterns, depending on the extension of the blue signal along the proximo-distal axis, indicated as distal (D), median–distal (MD), median–proximal (MP), and proximal (P). No GUS refers to those leaves in which the GUS staining has completely withdrawn from the blade (E). The numbers in the graphs indicate the percentage of leaves falling into each expression pattern, in the specified light conditions (F, G).

Mesophyll cell differentiation is associated with cessation of cell cycling which occurs from the leaf apex to the base. In fact, while dividing cells are initially distributed uniformly and diffusely throughout the leaf, cells near the blade apex cease dividing first and the region of frequent cell divisions gradually becomes restricted to the leaf base, forming a strong longitudinal gradient (Pyke *et al.*, 1991; Van Lijsebettens and Clarke, 1998; Donnelly *et al.*, 1999; Carabelli *et al.*, 2007; Kazama *et al.*, 2010; Andriankaja *et al.*, 2012). Thus, to investigate whether the differentiation process is indeed initiated earlier in shade, we analysed the changes in the spatial pattern of cell cycling during leaf expansion in High R/FR_High PAR_ and Low R/FR_Low PAR_ using the widely utilized CYCB1;1:GUS marker (Donnelly *et al.*, 1999). To this end, CYCB1;1:GUS first and second leaves of different ages but the same area in High R/FR_High PAR_ (7.5- and 8.5-day-old seedlings) and Low R/FR_Low PAR_ (9- and 10-day-old seedlings) were analysed. The histochemical localization of GUS activity in the adaxial subepidermal cell layer revealed that CYCB1;1:GUS spatial patterns differ in High R/FR_High PAR_ and Low R/FR_Low PAR_, and indicated that cell cycling of mesophyll precursors is terminated earlier in Low R/FR_Low PAR_ than in High R/FR_High PAR_ (Fig. 2A–D). To perform a quantitative analysis, first and second leaves were grouped into four GUS expression patterns indicated as distal (D), distal–median (DM), median–proximal (MP), and proximal (P), and the percentage of leaves showing each specific GUS pattern was determined (Fig. 2E–G). A large percentage of leaves with an area of ~0.25 mm^2^ (see legend to Fig. 2 for mean leaf area) displayed an MP and P pattern in High R/FR_High PAR_ and Low R/FR_ow PAR_, respectively (no. of leaves High R/FR_High PAR_=13/19; no. of leaves Low R/FR_Low PAR_=21/32; Fig. 2A, B, E, F). Furthermore, the totality of leaves with an area of ~0.50 mm^2^ (see legend to Fig. 2 for mean leaf area) had a P pattern in High R/FR_High PAR_ and no GUS labelling in Low R/FR_Low PAR_ (no. of leaves High R/FR_High PAR_=10/10; no. of leaves Low R/FR_Low PAR_=11/11; Fig. 2C, D, E, G). Consistent with the CYCB1;1:GUS spatial patterns, precursors mesophyll cells are larger in Low R/FR_Low PAR_ than in High R/FR_High PAR_ (Supplementary Table S2).

Together, the data indicate that prolonged shade provokes early differentiation of mesophyll cells which correlates with a precocious termination of vein formation.

### The HD-Zip transcription factor ATHB2 is required for early exit from proliferation during leaf development in shade

**Fig. 3.**
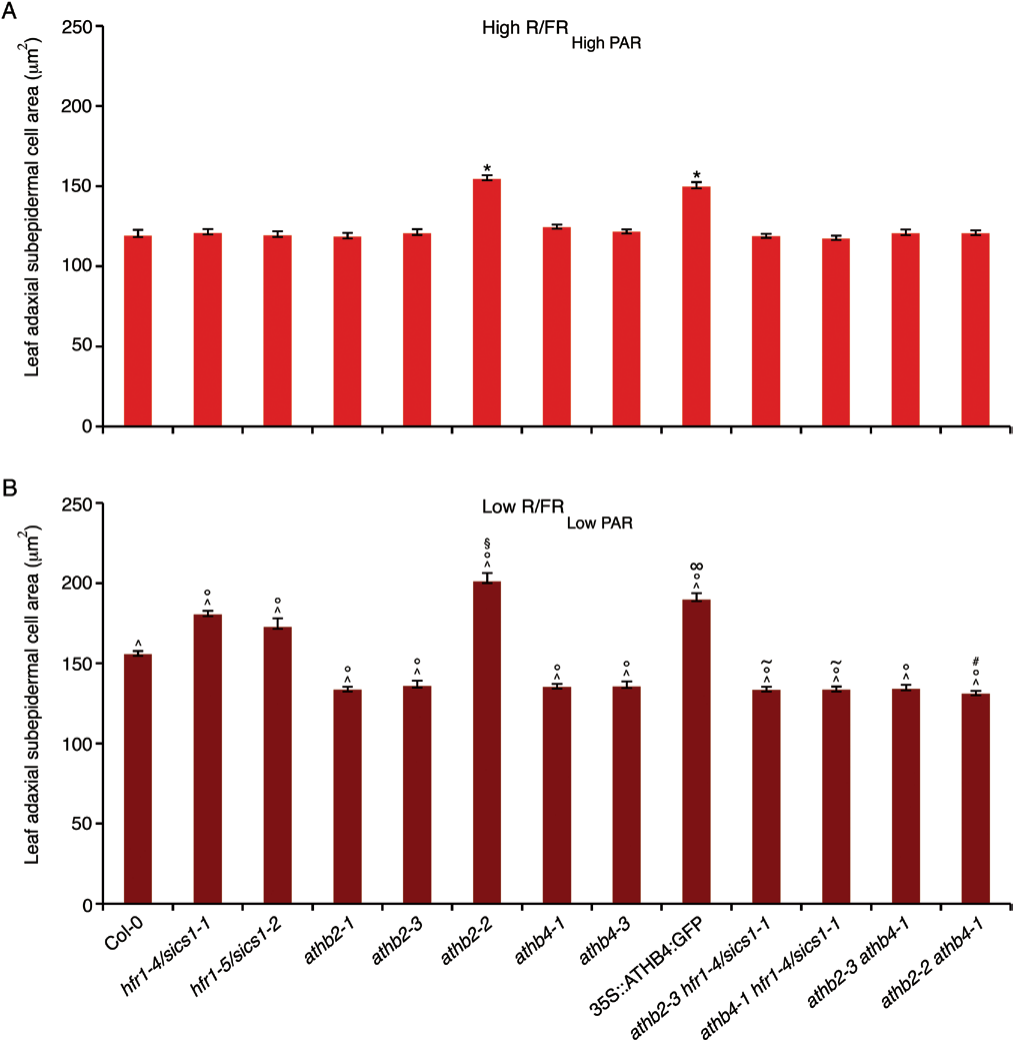
Leaf cell response to shade requires the HD-Zip II proteins ATHB2 and ATHB4. Col-0, *hfr1-4/sics1-1*, *hfr1-5/sics1-2*, *athb2-1*, *athb2-3*, *athb2-2*, *athb4-1*, *athb4-3*, 35S::ATHB4:GFP#13-3, *athb2-3 hfr1-4/sics1-1*, *athb4-1 hfr1-4/sics1-1*, *athb2-3 athb4-1*, and *athb2-2 athb4-1* seedlings were grown in a light/dark cycle (16/8 h) in High R/FR_High PAR_ (8 d for all the lines) (High R/FR_High PAR_), or for 4 d in high R/FR and subsequently for 5.5 d (Col-0, *athb2-1*, *athb2-3*, *athb2-2, athb4-1*, *athb4-3*, *athb2-3 athb4-1*, *athb2-2 athb4-1*) or 6.5 d (*hfr1-4/sics1-1*, *hfr1-5/sics1-2*, 35S::ATHB4:GFP#13-3, *athb2-3 hfr1-4/sics1-1*, *athb4-1 hfr1-4/sics1-1*) in Low R/FR_Low PAR_ (Low R/FR_Low PAR_). The graph shows the mean area of adaxial subepidermal cells of wild-type, mutant, and transgenic leaves with an area of ~0.35 mm^2^ in High R/FR_High PAR_ and Low R/FR_Low PAR_. First and second leaf area (mean ±SE), High R/FR_High PAR_: Col-0, 0.35 ± 0.004 mm^2^; *hfr1-4/sics1-1*, 0.35 ± 0.005 mm^2^; *hfr1-5/sics1-2*, 0.34 ± 0.008 mm^2^; *athb2-1*, 0.35 ± 0.003 mm^2^; *athb2-3*, 0.35 ± 0.005 mm^2^; *athb2-2*, 0.35 ± 0.003 mm^2^; *athb4-1*, 0.35 ± 0.006 mm^2^; *athb4-3*, 0.34 ± 0.003 mm^2^; 35S::ATHB4:GFP#13-3, 0.33 ± 0.003 mm^2^; *athb2-3 hfr1-4/sics1-1*, 0.35 ± 0.005 mm^2^; *athb4-1 hfr1-4/sics1-1*, 0.35 ± 0.004 mm^2^; *athb2-3 athb4-1*, 0.34 ± 0.003 mm^2^; *athb2-2 athb4-1*, 0.33 ± 0.006 mm^2^. First and second leaf area (mean ±SE), Low R/FR_Low PAR_: Col-0, 0.35 ± 0.003 mm^2^; *hfr1-4/sics1-1*, 0.34 ± 0.005 mm^2^; *hfr1-5/sics1-2*, 0.34 ± 0.009 mm^2^; *athb2-1*, 0.34 ± 0.004 mm^2^; *athb2-3*, 0.36 ± 0.008 mm^2^; *athb2-2*, 0.34 ± 0.007 mm^2^; *athb4-1*, 0.35 ± 0.007 mm^2^; *athb4-3*, 0.35 ± 0.005 mm^2^; 35S::ATHB4:GFP#13-3, 0.36 ± 0.003 mm^2^; *athb2-3 hfr1-4/sics1-1*, 0.34 ± 0.005 mm^2^; *athb4-1 hfr1-4/sics1-1*, 0.35 ± 0.004 mm^2^; *athb2-3 athb4-1*, 0.35 ± 0.004 mm^2^; *athb2-2 athb4-1*, 0.35 ± 0.008 mm^2^. *P<0.0001 *athb2-2*, 35S::ATHB4:GFP#13-3 High R/FR_High PAR_ versus Col-0 High R/FR_High PAR_; ^*P*<0.0001 Col-0, *hfr1-4/sics1-1*, *hfr1-5/sics1-2*, *athb2-1*, *athb2-3*, *athb2-2*, *athb4-1*, *athb4-3,* 35S::ATHB4:GFP#13-3, *athb2-3 hfr1-4/sics1-1*, *athb4-1 hfr1-4/sics1-1*, *athb2-3 athb4-1*, *athb2-2 athb4-1* Low R/FR_Low PAR_ versus Col-0 High R/FR_High PAR_; °*P*<0.0001 *hfr1-4/sics1-1*, *hfr1-5/sics1-2*, *athb2-1*, *athb2-3*, *athb2-2, athb4-1*, *athb4-3*, 35S::ATHB4:GFP#13-3, *athb2-3 hfr1-4/sics1-1*, *athb4-1 hfr1-4/sics1-1*, *athb2-3 athb4-1*, *athb2-2 athb4-1* Low R/FR_Low PAR_ versus Col-0 Low R/FR_Low PAR_; §*P*<0.0001 *athb2-2* Low R/FR_Low PAR_ versus *athb2-2* High R/FR_High PAR_; ∞*P*<0.0001 35S::ATHB4:GFP#13-3 Low R/FR_Low PAR_ versus 35S::ATHB4:GFP#13-3 High R/FR _High PAR_; ~*P*<0.0001 *athb2-3 hfr1-4/sics1-1*, *athb4-1 hfr1-4/sics1-1* Low R/FR_Low PAR_ versus *hfr1-4/sics1-1* Low R/FR_Low PAR_; # *P*<0.0001 *athb2-2 athb4-1* Low R/FR_Low PAR_ versus *athb2-2* Low R/FR_Low PAR._

Consistent with HFR1/SICS1 acting as a negative master regulator of the shade avoidance response (Sessa *et al.*, 2005; Hornitschek *et al.*, 2009), the leaf cell phenotype is exaggerated in *hfr1/sics1* loss-of-function mutant seedlings in Low R/FR_Low PAR_ (Fig. 3). No significant difference was observed between wild-type and *hfr1/sics1* leaves with an area of ~0.35 mm^2^ in High R/FR_High PAR_. In contrast, both *hfr1-4/sics1-1* and *hfr1-5/sics1-2* display precursor mesophyll cells larger than the wild type in leaves with an area of ~0.35 mm^2^ in Low R/FR_Low PAR_ (Fig. 3).

Among the genes up-regulated in *hfr1/sics1* mutants is *ATHB2* (Sessa *et al.*, 2005; Ruzza *et al.*, 2014), which has been suggested to play a role, together with *HAT1* and *HAT2*, in the control of cell proliferation during leaf development in a sun-simulated environment (Ciarbelli *et al.*, 2008).

**Fig. 4.**
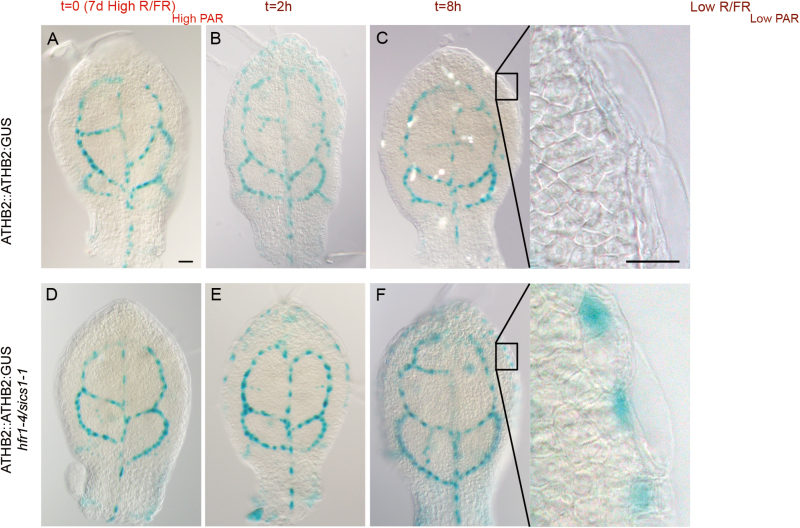
ATHB2 expression is induced in the leaf margin by shade. (A–F) Histochemical localization of GUS activity in first and second leaves of ATHB2::ATHB2:GUS (A–C) and ATHB2::ATHB2:GUS *hfr1-4/sics1-1* (D–F) seedlings grown for 7 d in a light/dark cycle (16/8 h) in High R/FR_High PAR_ (A, D), and then exposed to Low R/FR_Low PAR_ under the same regimen for 2 h (B, E) and 8 h (C, F). The insets show a paradermal view of adaxial epidermal cells. At least 30 leaves for each line and each time point were analysed. Scale bars: (A–F), 50 μm; insets, 10 μm.

Under a sun-simulated environment, ATHB2 expression is mainly localized in provascular cells in either the embryo or leaf primordia (Turchi *et al.*, 2013). Light quality changes rapidly, and transiently induces ATHB2:GUS expression in all cell layers of the elongating portion of the hypocotyl and cotyledon petioles of young seedlings (Carabelli *et al.*, 2013; Ruzza *et al.*, 2014). Consistent with HFR1/SICS1 function in shade avoidance, ATHB2::ATHB2:GUS is significantly up-regulated in the hypocotyl and cotyledon petioles of *hfr1-4/sics1-1* loss-of-function mutant seedlings upon prolonged exposure to simulated shade (Ruzza *et al.*, 2014). To determine whether ATHB2 is regulated by shade in leaves as well, ATHB2::ATHB2:GUS and *hfr1-4/sics1-1* ATHB2::ATHB2:GUS seedlings were grown for 7 d in High R/FR_High PAR_, and then transferred to Low R/FR_Low PAR_ for different times. Interestingly, ATHB2:GUS expression is transiently induced by shade in the wild type at the leaf margin (Fig. 4A–C), which is known to play a significant role in leaf development (Reinhardt *et al.*, 2007; Bilsborough *et al.*, 2011). In accordance with HFR1/SICS1 function in plant response to shade, the GUS signal in epidermal cells was stronger in *hfr1-4/sics1-1* ATHB2::ATHB2:GUS than in ATHB2::ATHB2:GUS leaves exposed to prolonged Low R/FR_Low PAR_ (Fig. 4D–F, insets).

To investigate whether ATHB2 has a role in the early exit from proliferation during leaf development in shade, the leaf cell phenotype of *athb2* loss- and gain-of-function mutants was analysed. Both *athb2-1* and *athb2-3* loss-of-function mutants display precursor mesophyll cells smaller than the wild type in leaves with an area of ~0.35 mm^2^ in Low R/FR_Low PAR_, whereas no significant difference was observed between mutants and the wild type in High R/FR_High PAR_ (Fig. 3). In contrast, the *athb2-2* gain-of-function mutant displays precursor mesophyll cells larger than the wild type in both High R/FR_High PAR_ and Low R/FR_Low PAR_ (Fig. 3).

**Fig. 5.**
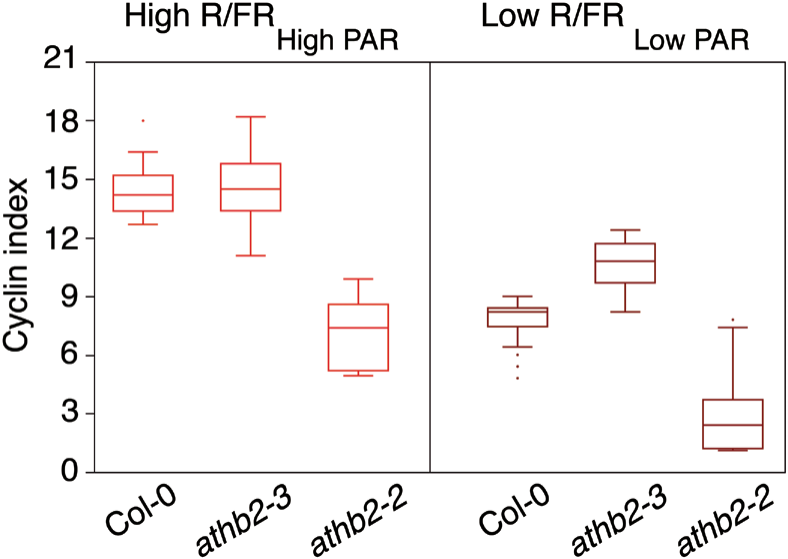
Loss- and gain-of-function mutations in *ATHB2* affect the expression of the CYCB1;1:GUS marker during leaf development in shade. CYCB1;1:GUS, *athb2-3* CYCB1;1:GUS, and *athb2-2* CYCB1;1:GUS seedlings were grown in a light/dark cycle (16/8 h) in High R/FR_High PAR_ for 8 d (High R/FR_High PAR_) or for 4 d in high R/FR and subsequently for 5.5 d in Low R/FR_Low PAR_ (Low R/FR_Low PAR_). The box-and-whiskers plot presents the cyclin indices for the adaxial subepidermal layer in the proximal region of wild-type and mutant leaves with an area of ~0.35 mm^2^ under the two different light conditions. The box delimits the first and third quartiles; the solid line within the box represents the second quartile; bars indicate the upper and lower fence; dots represent outliers. First and second leaf area (mean ±SE), High R/FR_High PAR_: CYCB1;1:GUS, 0.35 ± 0.004 mm^2^; *athb2-3* CYCB1;1:GUS, 0.35 ± 0.003 mm^2^; *athb2-2* CYCB1;1:GUS, 0.35 ± 0.003 mm^2^. First and second leaf area (mean ±SE), Low R/FR_Low PAR_: CYCB1;1:GUS, 0.35 ± 0.004; *athb2-3* CYCB1;1:GUS, 0.35 ± 0.003 mm^2^; *athb2-2* CYCB1;1:GUS, 0.35 ± 0.004 mm^2^.

To gain further insights into the role of ATHB2 in leaf development in shade, the CYCB1;1:GUS marker was introgressed into *athb2-2* and *athb2-3* mutant plants, and the cyclin index in the adaxial subepidermal cell layer of leaves with an area of ~0.35 mm^2^ in High R/FR_High PAR_ and Low R/FR_Low PAR_ was calculated (Donnelly *et al.*, 1999). As expected from leaf cell size analysis, the cyclin index value is higher in the leaf proximal region of *athb2-3* upon exposure to Low R/FR_Low PAR_ relative to the wild type, whereas it is lower in the *athb2-2* gain-of-function mutant (Fig. 5).

Together, the data demonstrate a role for ATHB2 in the control of cell proliferation during leaf development in shade. This is further supported by the finding that at the end of vegetative development, fully expanded first leaves are significantly larger in the *athb2* loss-of-function mutant than in the wild type in Low R/FR_Low PAR_ (Col-0, mean leaf area 6.01 ± 0.11 mm^2^; *athb2-3*, mean leaf area 6.59 ± 0.13 mm^2^; *P*<0.001) and not in High R/FR_High PAR_ (Col-0, mean leaf area 13.02 ± 0.26 mm^2^; *athb2-3*, mean leaf area 12.91 ± 0.27 mm^2^). No significant difference was observed in mesophyll cell area between wild-type and mutant leaves in High R/FR_High PAR_ and Low R/FR_Low PAR_. It can therefore be concluded that, under our experimental conditions, the leaf size difference between High R/FR_High PAR_ and Low R/FR_Low PAR_ is largely determined by cell number in a process partly controlled by ATHB2.

### ATHB2 and ATHB4 act together in controlling early exit from proliferation during leaf development in shade

Adaxial subepidermal cell size analysis and cyclin index determination of leaves with an area of ~0.35 mm^2^ (see Figs 3 and 5) demonstrated that the leaf response to Low R/FR_Low PAR_ is strongly reduced but not abolished in plants lacking ATHB2 function, thus indicating that other factor(s) are also involved in the early exit from proliferation provoked by canopy shade.

**Fig. 6.**
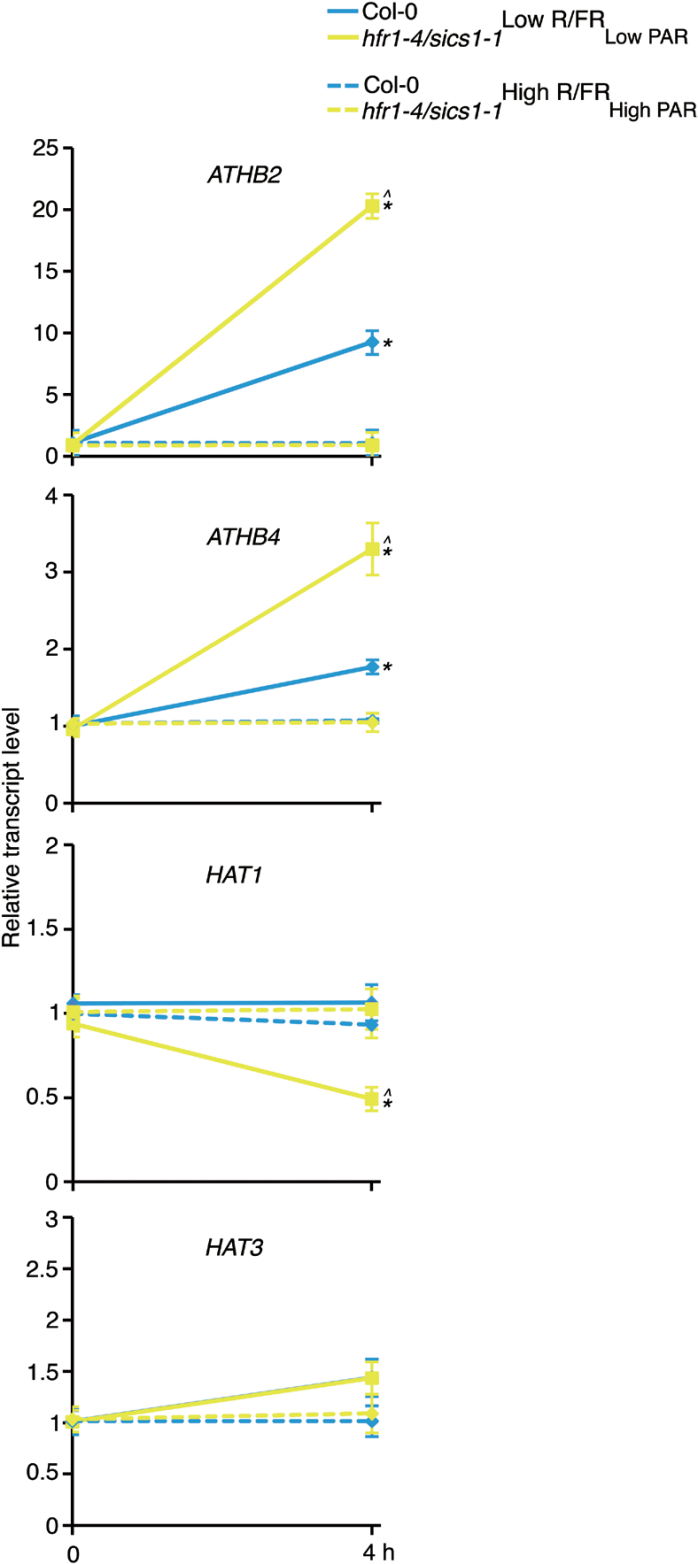
The HD-Zip II *ATHB4* gene is up-regulated in *hfr1/sics1* mutant seedlings in shade. RT-qPCR analyses of *ATHB2*, *ATHB4*, *HAT1*, and *HAT3* in Col-0 and *hfr1-4/sics1-1*. Plants were grown for 7 d in a light/dark cycle (16/8 h) in High R/FR_High PAR_, and then either maintained in High R/FR_High PAR_ (High R/FR_High PAR_) or transferred to Low R/FR_Low PAR_ (Low R/FR_Low PAR_) under the same regimen for 4 h. Plant transfer to Low R/FR_Low PAR_ was performed 4 h after the beginning of the light period. The graphs show the relative expression levels in High R/FR_High PAR_ and Low R/FR_Low PAR_ of *ATHB2*, *ATHB4*, *HAT1*, and *HAT3* in the two genotypes. Each value is the mean of three biological replicates normalized to *EF1α* expression (±SD). Statistical significance was assessed by means of one-way ANOVA followed by Tukey’s test. *P<0.001 Col Low R/FR_Low PAR_ and *hfr1-4/sics1-1* Low R/FR_Low PAR_ versus Col-0 High R/FR_High PAR_; ^*P*<0.001 *hfr1-4/sics1-1* Low R/FR_Low PAR_ versus Col-0 Low R/FR_Low PAR_.

Functional redundancy has been reported for members of the HD-Zip II γ and δ subfamilies (Ciarbelli *et al.*, 2008; Turchi *et al.*, 2013), suggesting that one or more HD-Zip II protein may contribute, together with ATHB2, to the early exit from proliferation during leaf development in shade. As a first step to test this, we investigated how a *hfr1/sics1* loss-of-function mutation affects the expression of *ATHB4*, *HAT1*, and *HAT3* in Low R/FR_Low PAR_. To this end, Col-0 and *hfr1-4/sics1-1* seedlings were grown for 7 d in a light/dark cycle in simulated sun, and then either maintained in the same regimen (High R/FR_High PAR_) or exposed to Low R/FR_Low PAR_ for 4 h (Low R/FR_Low PAR_). *ATHB-4*, like *ATHB2*, is significantly up-regulated in the *hfr1-4/sics1-1* loss-of-function mutant relative to the wild type in Low R/FR_Low PAR_, whereas *HAT1* and *HAT3* are not (Fig. 6).

To explore whether ATHB4 has any role in the leaf cell response to prolonged shade, the leaf cell phenotype of *athb4* loss-of-function mutants was characterized. Both *athb4-1* and *athb4-3* display precursor mesophyll cells smaller than the wild type in Low R/FR_Low PAR_, whereas no significant difference was observed between *athb4* mutants and the wild type in High R/FR_High PAR_ (Fig. 3). As observed in seedlings lacking ATHB2, however, the adaxial subepidermal cells of *athb4* mutant leaves are larger in Low R/FR_Low PAR_ than in High R/FR_High PAR_ (Fig. 3), indicating that in the absence of ATHB4 the leaf response to prolonged shade is reduced but not abolished. In contrast, 35S::ATHB4:GFP leaves display precursor mesophyll cells larger than the wild type in both High R/FR_High PAR_ and Low R/FR_Low PAR_ (Fig. 3).

The up-regulation of *ATHB2* and *ATHB4* in *hfr1-4/sics1-1* in Low R/FR_Low PAR_ and the phenotype of *athb2* and *athb4* loss- and gain-of-function mutants strongly suggest that the exaggerated leaf cell phenotype of *hfr1/sics1* loss-of-function mutants in shade may be attributable at least in part to the high levels of *ATHB2* and *ATHB4* expression in this mutant. To test this, we isolated and characterized the *athb2-3 hfr1-4/sics1-1* and the *athb4-1 hfr1-4/sics1-1* double mutants. First, we analysed the expression levels of *ATHB2* and *ATHB4*, respectively, in *athb4-1 hfr1-4/sics1-1*, and *athb2-3 hfr1-4/sics1-1* upon exposure to Low R/FR_Low PAR_ and found that the two genes are indeed up-regulated in the double mutants relative to the wild type (Supplementary Fig. S2). Next, we investigated the leaf cell phenotype of *athb2-3 hfr1-4/sics1-1*, and *athb4-1 hfr1-4/sics1-1* upon prolonged exposure to simulated shade. Leaf adaxial subepidermal cells of *athb2-3 hfr1-4/sics1-1* and *athb4-1 hfr1-4/sics1-1* resemble, respectively, those of *athb2-3* and *athb4-1* single mutants in Low R/FR_Low PAR_ (Fig. 3), implying that the exaggerated leaf cell phenotype of *hfr1/sics1* in shade is indeed caused by elevated levels of ATHB2 and ATHB4. Interestingly, however, the leaf cell phenotype of the double mutants also indicates that the enhanced response of *hfr1/sics1* leaves is lost in the absence of either ATHB2 or ATHB4, thus suggesting that the two proteins work in concert. To gain further insights on this, the leaf cell phenotype of the *athb2-3 athb4-1* and *athb2-2 athb4-1* double mutants was analysed. Consistent with the two proteins acting together, the size of the adaxial subepidermal cells of the *athb2-3 athb4-1* double loss-of-function mutant exposed to prolonged Low R/FR_Low PAR_ does not differ from that of the single mutants, whereas the leaf cell phenotype of *athb2-2 athb4-1* resembles that of *athb4-1* in both High R/FR_High PAR_ and Low R/FR_Low PAR_ (Fig. 3). Thus, this imples that the leaf cell phenotype caused by the *athb2-2* gain-of-function mutation depends on the presence of ATHB4.

### Low PAR determines early exit from proliferation in the leaf through an ATHB2-independent pathway

Shade avoidance response is triggered by both changes in spectral composition and a reduction in the total amount of visible light. Recent work argues that there is more than one pathway leading to growth of the hypocotyl in response to changes in light quality [low R/FR or low blue light (LBL)] or quantity (PAR) (Keller *et al.*, 2011; Keuskamp *et al.*, 2011; Pedmale *et al.*, 2016). Under our simulated shade conditions, R is reduced and FR is increased, maintaining total light quantity (400–800 nm) constant (Sessa *et al.*, 2005; Carabelli *et al.*, 2007; Ciarbelli *et al.*, 2008; Ciolfi *et al.*, 2013). Varying R/FR while maintaining total light quantity constant implies a non-constant supply of PAR (400–700 nm). Consistent with the existence of more than one pathway promoting hypocotyl growth in response to light quality changes or low PAR, we have previously shown that there is no significant difference in the expression of several genes rapidly induced by shade such as *ATHB2* (Carabelli *et al.*, 1996), *PIL1* (Salter *et al.*, 2003), and *HFR1/SICS1* (Sessa *et al.*, 2005) between High R/FR_High PAR_ and High R/FR_Low PAR_, thus demonstrating that these genes are specifically regulated at the transcriptional level by light quality changes under our simulated shade environment (Ciolfi *et al.*, 2013).

The wild type exposed to High R/FR_Low PAR_ displays precursor mesophyll cells larger than those of seedlings grown in High R/FR_High PAR_ (Supplementary Fig. S3). To investigate whether ATHB2 has any role in this process, the leaf cell phenotype of *athb2-3*, *hfr1-4/sics1-1*, and *athb2-3 hfr1-4/sics1-1* was analysed. No significant difference was observed between mutants and the wild type in High R/FR_Low PAR_, thus indicating that low PAR affects leaf development through a pathway independent of ATHB2 (Supplementary Fig. S3).

## Discussion

### Effects of shade on leaf morphogenesis

Leaf growth is driven by two tightly controlled processes, cell proliferation and subsequent cell expansion which determine leaf size and shape (Gonzales *et al.*, 2012). These processes are dynamically regulated by environmental cues (Carabelli *et al.*, 2007; Skirycz and Inzé, 2010; Skirycz *et al.*, 2011; Kalve *et al.*, 2014). A number of studies have indicated that low light intensity reduces the leaf area in several plant species (Wilson, 1966; Dengler, 1980; Yano and Terashima, 2004; Kim *et al.*, 2005; Cookson and Granier, 2006). It has been suggested that a reduction in cell number, not in cell size, contributes to the reduced leaf size of plants grown under a simulated shade light regime consisting of low R/FR and low photosynthetic light radiation (Low R/FR_Low PAR_) (Carabelli *et al.*, 2007). Moreover, evidence has been provided that Low R/FR_Low PAR_ rapidly and transiently reduces the frequency of cell division in young leaf primordia (~0.008 mm^2^) through a non-cell-autonomous mechanism (Carabelli *et al.*, 2007, 2008; Ruzza *et al.*, 2014). Here we show that persistence of Low R/FR_Low PAR_, later during leaf development (>0.25 mm^2^), determines early exit from proliferation, which in turn results in smaller leaves at the end of vegetative development. By combining adaxial subepidermal cell size determination and the expression pattern of the CYCB1;1:GUS marker in the first and second leaves of plants grown in High R/FR_High PAR_ and Low R/FR_Low PAR_, we indeed gained evidence that prolonged exposure to simulated shade provokes early termination of cell cycling of mesophyll precursors (Figs 1, 2; Supplementary Table S2). Furthermore, taking advantage of the ATHB8::GUS marker (Baima *et al.*, 1995), we demonstrated that the early differentiation of leaf mesophyll cells in plants grown in Low R/FR_Low PAR_ relative to those in High R/FR_High PAR_ correlates with a precocious termination of vein formation (Fig. 1). This finding is consistent with the evidence that Arabidopsis vein pattern is not inherently determinate, but arises through reiterative initiation of new pre-procambial branches until this process becomes terminate by the differentiation of mesophyll (Scarpella *et al.*, 2004).

Very recently it has been shown that soybean plants grown in shade produced smaller leaves as a result of a lower mitotic activity. These results are also corroborated by a lower expression of key genes involved in the regulation of cell proliferation. In addition, shade significantly increased the auxin and gibberellin content, and significantly decreased the cytokinin (CK) content in soybean leaves. (Wu *et al.*, 2017). Several studies pointed to auxin as a major player in shade avoidance response and in neighbour detection in Arabidopsis and crop species (Casal, 2013; Iglesias *et al.*, 2018, and reference therein). We have previously shown that shade light triggers a rapid arrest of leaf primordia growth by the breakdown of CKs through the action of the auxin-induced cytokinin oxidase 6 gene (*AtCKX6*) (Carabelli *et al.*, 2007). Moreover, long exposure to shade light results in an up-regulation of *AtCKX5* (Ciolfi *et al.*, 2013). Considering the positive role of CK in the regulation of cell proliferation in the shoot (Kieber and Schaller, 2018) and the negative effects of an increased CK degradation on leaf size (Holst *et al.*, 2011), it is tempting to speculate that a reduced CK signalling could be responsible in part for the smaller leaves found in plants grown under shade light.

A rapid and transient arrest in cell cycle progression (pause) has also been observed in young Arabidopsis leaves subjected to mild osmotic stress (Skirycz *et al.*, 2011). Interestingly, Skirycz and co-authors (2011) in addition found that when the osmotic stress persists, cells exit the mitotic cell cycle and initiate the differentiation process (stop) (Skirycz *et al.*, 2011). These data together with those observed under simulated shade (Carabelli *et al.*, 2007; this work) lead to speculation that ‘pause-and-stop’ may be a general mechanism by which plants respond to environmental conditions suboptimal for growth.

### Role of HD-ZIP II transcription factors in the morphogenesis of shade leaves

In the attempt to identify the regulatory gene(s) involved in the early exit of leaf adaxial subepidermal cells from proliferation in plants grown under simulated shade, we searched for genes up-regulated in *hfr1/sics1* loss-of-function mutant seedlings in Low R/FR_Low PAR_, which, in accordance with HFR1/SICS1 acting as a negative master regulator of the shade avoidance response (Sessa *et al.*, 2005; Hornitschek *et al.*, 2009), display an exaggerated leaf cell phenotype. Among them is *ATHB2* (Sessa *et al.*, 2005; Ruzza *et al.*, 2014), a HD-Zip II transcription factor gene, which has been suggested to play a role, together with the HD-Zip II genes *HAT1* and *HAT2*, in the control of cell proliferation during leaf development in a sun-simulated environment (Ciarbelli *et al.*, 2008). It has indeed been observed that the mean area of leaf adaxial subepidermal cells of plants overexpressing ATHB2, HAT1, or HAT2 was significantly larger than that of the wild type in High R/FR_High PAR_. In contrast, overexpression of a dominant negative derivative of ATHB2 (ATHB2N51A), thought to sequester the endogenous ATHB2 protein and, probably, related HD-Zip II proteins in functionally inactive heterodimeric complexes, resulted in a decrease of the mean area of leaf adaxial subepidermal cells relative to the control (Ciarbelli *et al.*, 2008). Remarkably, by combining adaxial subepidermal cell size determination and cyclin index calculation in the first and second leaves of plants grown in High R/FR_High PAR_ and Low R/FR_Low PAR_, here we show that loss of function of *ATHB2* delays exit from proliferation relative to the wild type under simulated shade, whereas gain of function of *ATHB2* causes early termination of cell cycling of mesophyll precursors under both light regimes, thus implying a major role for ATHB2 in leaf development during shade avoidance (Figs 3, 5). Consistent with this, at the end of vegetative development, fully expanded first leaves were significantly larger in the *athb2* loss-of function mutant than in the wild type in Low R/FR_Low PAR_ and not in High R/FR_High PAR_, whereas no significant difference was observed in mesophyll cell area between wild-type and mutant leaves in High R/FR_High PAR_ and Low R/FR_Low PAR._ This is particularly significant considering the potential to recover the decreased cell number through the activity of meristemoids (Skirycz *et al.*, 2011).

Among the genes up-regulated in *hfr1/sics1* loss-of-function mutants is also *ATHB4*, a gene encoding a HD-Zip II protein closely related to ATHB2 (Fig. 6). Interestingly, *athb4* loss-of-function mutants in Low R/FR_Low PAR_ display the same leaf cell phenotype observed in plants lacking ATHB2 grown under simulated shade (Fig. 3). In contrast, leaves of plants overexpressing ATHB4 display early exit from proliferation in both High R/FR_High PAR_ and Low R/FR_Low PAR_ with respect to the wild type (Fig. 3). Together, the data indicate that ATHB4 also contributes to leaf development during shade avoidance.

Intriguingly, *athb2 hfr1/sics1* and *athb4 hfr1/sics1* loss-of-function double mutants display a leaf cell phenotype in Low R/FR_Low PAR_ analogous to that of *athb2* and *athb4* single mutants grown under simulated shade, respectively (Fig. 3). This further indicates a major role for ATHB2 and ATHB4 in leaf development during shade avoidance. Moreover, considering that *ATHB2* and *ATHB4* are up-regulated in *athb4 hfr1/sics1* and *athb2 hfr1/sics1* double mutants upon exposure to Low R/FR_Low PAR_ relative to the wild type (Supplementary Fig. S2), respectively, the leaf cell phenotype of the double mutants under simulated shade indicates that the exaggerated response of *hfr1/sics1* leaves is lost in the absence of either ATHB2 or ATHB4, thus suggesting that the two proteins work in concert. Consistent with this, the leaf cell phenotype of the *athb2-3 athb4-1* double loss-of-function mutant grown in Low R/FR_Low PAR_ resembles that of the single *athb2-3* and *athb4-1* mutants (Fig. 3). Furthermore, remarkably, the phenotype of the *athb2* gain-of-function mutant is lost in plants lacking ATHB4 (Fig. 3), thus implying that the early termination of cell cycling of mesophyll precursors under both High R/FR_High PAR_ and Low R/FR_Low PAR_ caused by the *athb2-2* mutation depends on the presence of ATHB4. These data further indicate that ATHB2 and ATHB4 proteins function as a complex in the regulation of leaf development during shade avoidance. They probably form heterodimers as suggested by yeast two-hybrid assays (Trigg *et al.*, 2017).

A number of leaf growth regulators have been identified, and connections between these regulators have started to emerge (Gonzales *et al.*, 2012; Bar and Ori, 2014); some of these factors are involved in the regulation of leaf polarity and cell proliferation (Husbands *et al.*, 2015). Intriguingly, members of the HD-Zip II family, including ATHB2 and ATHB4, have been shown to regulate leaf polarity as well as shade avoidance response (Steindler *et al.*, 1999; Sorin *et al.*, 2009; Bou-Torrent *et al.*, 2012; Turchi *et al.*, 2013, 2015; Paz *et al.*, 2016). Understanding how ATHB2 and ATHB4 interact with known regulators affecting leaf proliferation will be essential to unravel the mechanisms underlying leaf development under canopy shade.

## Supplementary data

Supplementary data are available at *JXB* online.

Fig. S1. Kinetics of first and second leaf growth in High R/FR_High PAR_ and Low R/FR_Low PAR_.

Fig. S2. *ATHB2* and *ATHB4* genes are up-regulated respectively in *athb4-1 hfr1-4/sics1-1* and *athb2-3 hfr1-4/sics1-1* double mutants in Low R/FR_Low PAR_.

Fig. S3. Leaf phenotype of Col-0, *athb2-3*, *hfr1-4/sics1-1*, and *athb2-3 hfr1-4/sics1-1* seedlings in High R/FR_High PAR_ and High R/FR_Low PAR._

Table S1. Primers and UPL probes for RT-qPCR analyses.

Table S2. Leaf adaxial subepidermal cells are larger in Low R/FR _Low PAR_ than in High R/FR _High._

Supplementary Figure and Tables
