# Supplementary material for: Arabidopsis HD-Zip II proteins regulate the exit from proliferation during leaf development in canopy shade
Source: J Exp Bot. 2018 Sep 15;69(22):5419–31. doi: 10.1093/jxb/ery331 (PMC6255710; doi:10.1093/jxb/ery331)
Supplement: Supplementary Figure and Tables [file ery331_suppl_supplementary_figure_tables.pdf]

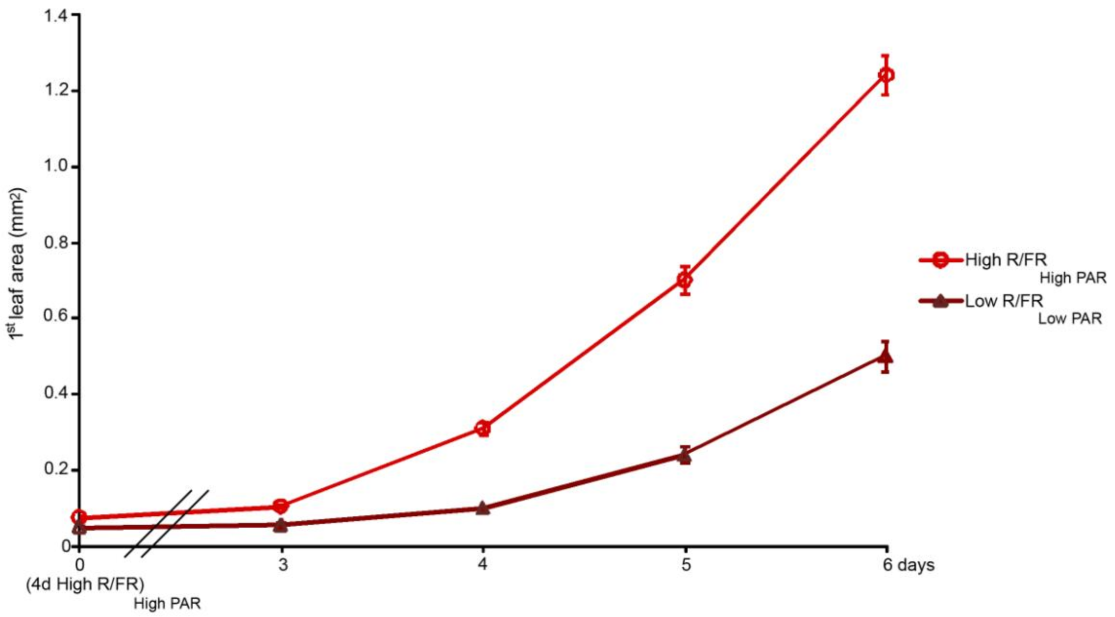

Supplementary Fig. S1. Kinetics of 1st-2nd leaf growth in High R/FR $_{\text{High PAR}}$ and Low R/FR $_{\text{Low PAR}}$. Col-0 seedlings were grown in a L/D cycle (16/8h) for 4 days in High R/FR $_{\text{High PAR}}$ (0), and subsequently maintained in High R/FR $_{\text{High PAR}}$ (High R/FR $_{\text{High PAR}}$) or transferred to Low R/FR $_{\text{Low PAR}}$ (Low R/FR $_{\text{Low PAR}}$) for the indicated times. The mean area of the 1st-2nd leaf was determined measuring 50 samples for each time point in High R/FR $_{\text{High PAR}}$ and Low R/FR $_{\text{Low PAR}}$.

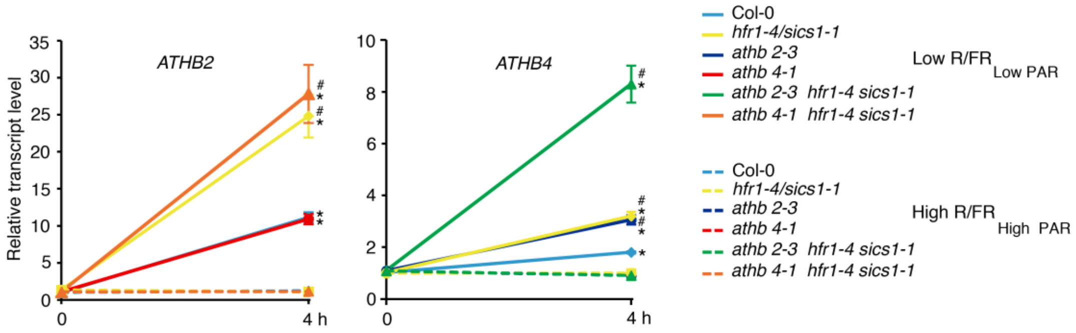

Supplementary Fig. S2. *ATHB2* and *ATHB4* genes are up-regulated respectively in *athb4-1 hfr1-4/sics1-1* and *athb2-3 hfr1-4/sics1-1* double mutants in Low R/FR $_{\text{Low PAR}}$. RT-qPCR analyses of *ATHB2* and *ATHB4* in Col-0, *athb2-3, athb4-1, hfr1-4/sics1, athb4-1 hfr1-4/sics1-1* and *athb2-3 hfr1-4/sics1-1*. Plants were grown for 7 days in a L/D cycle (16/8h) in High R/FR $_{\text{High PAR}}$, and then either maintained in High R/FR $_{\text{High PAR}}$ (High R/FR $_{\text{High PAR}}$) or transferred to Low R/FR $_{\text{Low PAR}}$ (Low R/FR $_{\text{Low PAR}}$) under the same L/D regimen for 4 hours. Plant transfer to Low R/FR $_{\text{Low PAR}}$ was performed 4 hours after the beginning of the light period. The graphs show the relative expression levels in High R/FR $_{\text{High PAR}}$ and Low R/FR $_{\text{Low PAR}}$ of *ATHB2* and *ATHB4* in the different genotypes. Each value is the mean of three biological replicates normalized to *EF1α* expression (±SD). Statistical significance was assessed by mean of one-way ANOVA analysis followed by Tukey's test. *P<0.001 Col-0, *hfr1-4/sics1-1*, *athb4-1*, *athb2-3*, *athb4-1 hfr1-4/sics1-1* and *athb2-3 hfr1-4/sics1-1* Low R/FR $_{\text{Low PAR}}$ *vs.* Col-0 High R/FR $_{\text{High PAR}}$; #P<0.001 *hfr1-4/sics1-1*, *athb4-1 hfr1-4/sics1-1*, *athb2-3* and *athb2-3 hfr1-4/sics1-1* Low R/FR $_{\text{Low PAR}}$ *vs.* Col-0 Low R/FR $_{\text{Low PAR}}$.

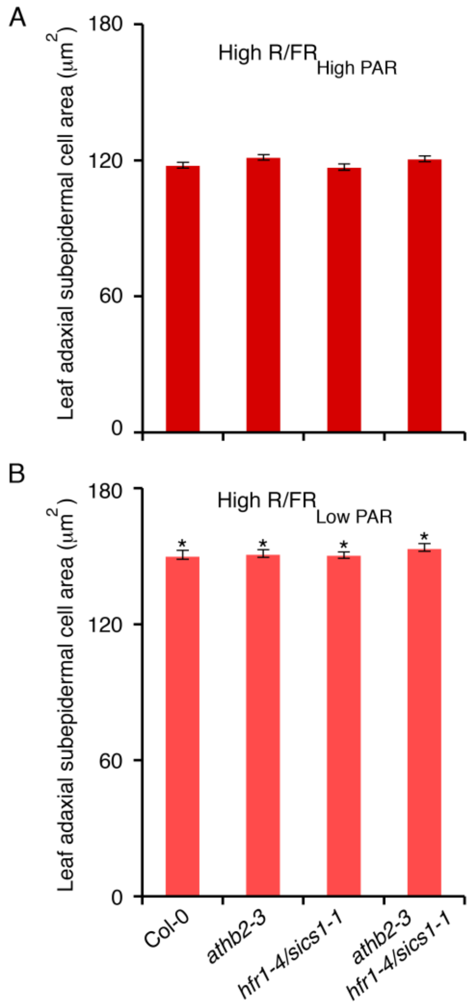

Supplementary Fig. S3. **Leaf phenotype of Col-0, *athb2-3, hfr1-4/sics1-1* and *athb2-3 hfr1-4/sics1-1* seedlings in High R/FR $_{\text{High PAR}}$ and High R/FR $_{\text{Low PAR}}$.** Col-0, *athb2-3*, *hfr1-4/sics1-1* and *athb2-3 hfr1-4/sics1-2* seedlings were grown in a L/D cycle (16/8h) in High R/FR $_{\text{High PAR}}$ for 8 days (High R/FR $_{\text{High PAR}}$) (A), or for 4 days in high R/FR and subsequently for 5.5 days in High R/FR $_{\text{Low PAR}}$ (High R/FR $_{\text{Low PAR}}$) (B). The graph shows the mean area of adaxial subepidermal cells of wild-type and mutant leaves with an area of approximately 0.35 mm^2 in High R/FR $_{\text{High PAR}}$ and High R/FR $_{\text{Low PAR}}$. 1st-2nd leaf area (mean ± s.e.m.), High R/FR $_{\text{High PAR}}$: Col-0, 0.33 ± 0.004 mm^2; *athb2-3*, 0.34 ± 0.004 mm^2; *hfr1-4/sics1-1*, 0.34 ± 0.005 mm^2; *athb2-3 hfr1-5/sics1-2* 0.34 ± 0.004 mm^2. 1st-2nd leaf area (mean ± s.e.m.), High R/FR $_{\text{Low PAR}}$: Col-0, 0.35 ± 0.007 mm^2; *athb2-3*, 0.34 ± 0.007 mm^2; *hfr1-4/sics1-1*, 0.34± 0.003 mm^2; *athb2-3 hfr1-4/sics1-2*, 0.34 ± 0,004 mm^2. *P<0.0001 Col-0, *athb2-3*, *hfr1-4/sics1-1*, *athb2-3 hfr1-4/sics1-2* High R/FR $_{\text{Low PAR}}$ *vs.* Col-0 High R/FR $_{\text{High PAR}}$.

Supplementary Table S1. Primers and UPL probes for RT-qPCR analyses

Gene	Primer 5’	Primer 3’	UPL
ATHB2	CAAATCCATCTGTTTCTGTTACTCC	TGTGACGAATCTGAGTTTGGA	#143
ATHB4	GCTTGAATCTTATGCCGTTGA	TGTGCATGTGTTGAAACGAA	#143
HAT1	AAACCCTTGTTTCTTCCTCAGA	GCTCGAGATCGTACTGTTTGG	#94
HAT3	GCATCTTCATCACACATGCAG	CGATCTCATGTCCGAGTTTCT	#84
EF1α	CTTGGTGTCAAGCAGATGATTT	CGTACCTAGCCTTGGAGTATTTG	#77

Supplementary Table S2. Leaf adaxial subepidermal cells are larger in Low R/FR $_{Low\ PAR}$ than in High R/FR $_{High\ PAR}$

		Cell area (μm^2±s.e.m.)	
1st-2nd Leaf ~0.25 mm^2	D	89.7±2.1	108.9±2.9*
	P	33.2±1.1	35.1±1.0
1st-2nd Leaf ~0.50 mm^2	D	189.1±4.3	205.2±4.0°
	P	35.2±0.9	50.6±1.3*
		High R/FR $_{High\ PAR}$	Low R/FR $_{Low\ PAR}$

CYCB1;1:GUS seedlings were grown on agar plates in a L/D cycle (16/8h) in High R/FR $_{High\ PAR}$ for 4 days and then maintained under the same light regime (High R/FR $_{High\ PAR}$) for further 3.5 and 4.5 days, or transferred to Low R/FR $_{Low\ PAR}$ (Low R/FR $_{Low\ PAR}$) for 5 and 6 days. 1st-2nd leaf area (mean ± s.e.m.): High R/FR $_{High\ PAR}$ ~0.25 mm^2, 0.25±0.011 mm^2; Low R/FR $_{Low\ PAR}$ ~0.25 mm^2, 0.24±0.009 mm^2; High R/FR $_{High\ PAR}$ ~0.50 mm^2, 0.53±0.013 mm^2; Low R/FR $_{Low\ PAR}$ ~0.50 mm^2, 0.54±0.012 mm^2.

At least 100 adaxial subepidermal cells in 10 leaves were measured for each condition.

D, distal; P, proximal.

*P<0.0001, °P<0.007 (Low R/FR $_{Low\ PAR}$ *vs.* High R/FR $_{High\ PAR}$).
